# Hydrogel Design Based on Bacterial Exopolysaccharides: The Biomedical Promise of Levan

**DOI:** 10.3390/ijms262210828

**Published:** 2025-11-07

**Authors:** Andrada Ioana Popa, Rahela Carpa, Anca Farkas

**Affiliations:** 1 Department of Molecular Biology and Biotechnology, Faculty of Biology and Geology, Babeș-Bolyai University, 1 M. Kogălniceanu Street, 400084 Cluj-Napoca, Romania; andrada.ioana.popa@stud.ubbcluj.ro (A.I.P.); ancuta.farkas@ubbcluj.ro (A.F.); 2 Institute for Research-Development-Innovation in Applied Natural Sciences, Babeș-Bolyai University, 30 Fântânele St., 400294 Cluj-Napoca, Romania

**Keywords:** hydrophilic gels, bacterial exopolysaccharides, levan, biomedical applications

## Abstract

Bacterial exopolysaccharides have emerged as versatile biopolymers for the design of advanced hydrogels with adjustable physico-chemical, mechanical, and biological properties. Among these, levan, a fructose-based exopolysaccharide synthesized by various microbial species, has attracted increasing attention due to its unique structural features, high biocompatibility, and inherent bioactivity. This review provides a comprehensive overview of hydrogel systems derived from bacterial exopolysaccharides, with a particular focus on levan-based hydrogels. We discuss the molecular structure, synthesis pathways, and physico-chemical characteristics of levan that underpin its hydrogel-forming ability. Emphasis is placed on design strategies, including chemical modification, crosslinking approaches, and composite formation, that enable fine-tuning of mechanical strength, swelling behavior, and degradation kinetics. This review further highlights biomedical applications of levan-based hydrogels, encompassing drug delivery, wound healing, rejuvenation, tissue engineering, regenerative medicine, and bioprinting, while addressing current limitations and future research directions. By elucidating the structure–function relationships and emerging fabrication methodologies, this review underscores the biomedical promise of levan as a sustainable and functional biopolymer for next-generation hydrogel technologies.

## 1. Introduction

Molecular aggregates formed by hydrophilic polymers and water molecules are biomaterials of major interest [1,2]. Natural compounds and synthetic analogs have been developed and modulated into various architectures to adapt to different applications [3,4,5].

Hydrophilic gels, frequently referred to as hydrogels, are tridimensional networks formed by cross-linked materials with a hydrophilic structure, capable of absorbing high quantities of water or biological fluids [6,7]. Due to the connections between the polymeric chains, the microscopic interactions and their density influence the behavior of the macroscopic molecule [7].

Depending on the source, composition, stimulus responsiveness, physical configuration, and also electronic charge, hydrogels can be classified as follows:

− Based on origin: natural or synthetic;
− Based on the polymers used in synthesis: homopolymeric—a singular repetitive cross-linked structural unit; copolymeric—two different monomers arranged in a random or alternate fashion in the polymeric network; and multipolymeric—a network of two cross-linked polymers and one non-linked monomer, consisting of either natural or synthetic molecules;
− Based on stimulus response: responsive at temperature, pH, ionic strength, and light- and chemical-responsive;
− Based on chemical structure: amorphous, semicrystalline, or crystalline;
− Based on electronic charge: nonionic, ionic, amphoteric, or zwitterionic (repetitions of cationic and anionic groups) [1,4,5,6].

In addition to an insight into the structure at a monomeric level, which forms linked or non-linked polymeric chains, a macromolecular approach to the structural particularities is required in order to infer their stability and potential applications [7]. Moreover, a key point resides in the understanding of the mechanisms behind the transition from a sol to a gel state [6].

A defining feature of hydrophilic gels is represented by the high content of absorbed water, which can surpass 95% of the mass ratio [1]. Another aspect resides in their adjustable porosity, which can be achieved through various techniques such as adjusting the polymer and cross-linker concentrations [8], freeze-drying [9], and cryogelation [10]. Other techniques include solvent casting/particle leaching, aided by the dispersion of porogen particles and leaching via selective solvents [11], or even gas foaming [12].

Due to their unique structure and properties, this group of molecular aggregates formed by hydrophilic polymers and water molecules is of major interest in materials science and engineering [13], with various applications in biomedicine [5,14], cosmetic industry [15], food processing [16], bioremediation technologies [17], and sustainable agriculture [18]. Various natural compounds and synthetic analogs have been recently developed. Their properties and, therefore, their stability can be modulated to adapt to certain media characterized by unfavorable conditions, such as significant pH variations, temperature, chemical stress, etc. [3,4,5].

Hydrogels provide an ideal platform for tridimensional cellular studies [6], being bioengineered for a wide range of biomedical applications, such as tissue engineering, bioprinting, and targeted drug delivery [1,2]. A number of studies have been conducted on such gels, exploiting their biomimetic properties, for example, the development of the extracellular matrix of pancreatic tissue [2], but also for cells of the vascular endothelium [10]. Areas pertaining to regenerative medicine and lesion treatment are indisputable, especially concerning infection control and maintenance of an optimal medium for hemostasis, inflammation, proliferation, and tissue remodeling [19]. Recent approaches focus on the creation of stimulus-responsive cellular constructs, especially in the context of regenerative medicine [1,2,6,20]. Incorporation of bioreceptors such as DNA, enzymes, antibodies, and even cells has led to the development of hydrogel-based sensors for the detection of small molecules, cellular metabolites, and pathogens. Biosensors and composite hydrogels are useful in metabolic diagnosis, cancer screening, and bacterial and viral sensing and capture [21,22,23].

The aim of this paper is to provide a comprehensive analysis of hydrogel design, with a special focus on levan as a promising bacterial exopolysaccharide for biomedical applications. This review integrates current knowledge and highlights emerging trends to emphasize levan-based hydrogels as sustainable, biocompatible, and multifunctional biomaterials for medical bioengineering.

## 2. Hydrophilic Gels Based on Bacterial Exopolysaccharides

Exopolysaccharides (EPSs) are a group of high-molecular-weight carbohydrate polymers [24] characterized by the presence of a variable number of functional groups, conferring both native properties and the possibility of molecular functionalization [25]. Depending on the repetitive structural unit, they can be classified as homoexopolysaccharides (same monomer linked through glycosidic bonds) and heteroexopolysaccharides (two or more monomers linked through glycosidic bonds) [25,26].

Hydrophilic gels based on bacterial EPSs are formed by cross-linking bacterial biopolymers with high affinity for water, like levan, dextran, cellulose, alginate, hyaluronic acid, gellan, kefiran, and xanthan gum [27,28,29]. These polysaccharides are usually secreted in the extracellular space, sometimes with a tight association with the cell wall, which facilitates scaling at the industrial level, but also higher efficacy for downstream processing [27,30]. The three-dimensional network of bacterial EPS gels is achieved through polymerization and self-assembly during physically or chemically driven cross-linking of biopolymers [31]. Hydrogels can be further modified or blended with other materials as composite hydrogels with enhanced mechanical strength and improved functionality [32,33].

The valuable properties of hydrogels based on bacterial EPSs are high water absorption, biodegradability without forming toxic byproducts, stability in different conditions, biocompatibility, conformational adaptability, the capacity of reverting to their initial state before having absorbed a biological fluid, and also stimulus responsiveness [4,6,19,29,32].

## 3. Bacterial Levan as a Structural Biopolymer for Hydrogels

Levan is a naturally occurring EPS diversely distributed in bacteria, fungi, and plants [34]. It is widely produced by a wide range of prokaryotes with diverse taxonomy: Gram-positive bacteria belonging to the phyla Actinomycetota and Bacillota, as well as Gram-negative bacteria belonging to Pseudomonadota [35]. Prokaryotic strains originating in a vast range of environments, from common natural settings to extreme habitats, plant or animal sources, as well as reference strains belonging to industrial culture collections, have been previously investigated for levan production. In addition, genomic analysis revealed putative levansucrase-encoding genes that allowed cloning, expression, and purification of enzymes for levan biosynthesis in recombinant or cell-free systems (Table 1).

**Figure 1:**
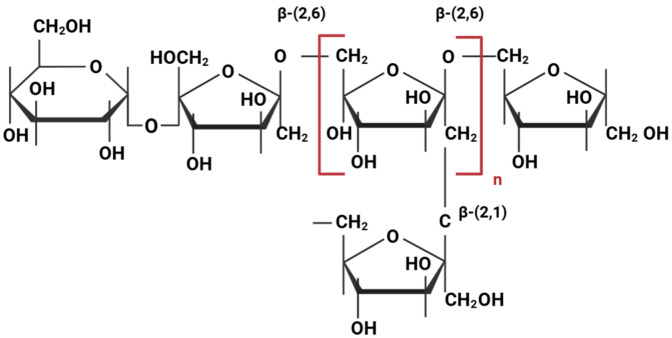
Chemical structure of levan.

The molecular structure of levan defines a homoexopolysaccharide with a backbone of fructose units linked through β-(2,6) glycosidic bonds, alongside a residual glucosyl molecule at the end of the polymeric chain. Short side chains consisting of fructose residues are attached to the main chain via β-(2,1) glycosidic bonds [24,34,66] (Figure 1). This branching is a key structural feature of levan, particularly in microbial levan, which can have a significantly higher molecular weight (28 to 141,000 kDa) and a greater degree of branching [67] compared to plant levan (2 to 33 kDa) [68]. The molecular weight determines polymer conformation and subsequent properties. Low-molecular-weight microbial-derived levans tend to form spherical nanoparticles, while high-molecular-weight levans exhibit microgel characteristics [67,69].

Levan biosynthesis differs significantly between bacteria and plants in terms of molecular weight, enzyme mechanisms, and location. Bacteria synthesize high-molecular-weight levan using enzymes like levansucrase (EC 2.4.1.10) to form a polymer with high viscosity, while plants produce low-molecular-weight levan, often within the vacuole [34]. In bacteria, prior to levan biosynthesis, levansucrase is synthesized in the cytoplasmic space; then, it is accumulated in the periplasm, where it undergoes conformational changes before its secretion through various pathways. Generally, this involves cleavage of a signal peptide in Gram-positive bacteria, yet the majority of Gram-negative bacteria have been described to secrete levansucrase in a signal peptide-independent pathway [70]. Moreover, the genes involved in levan biosynthesis are found in a tricistronic operon. Levansucrase is encoded by the gene *sacB*, while a protein with endolevanase activity is encoded by *yveB*. The function of the third protein, encoded by *yveA*, has not yet been determined, but reports have predicted that it might function as a permease [71,72].

**Figure 2:**
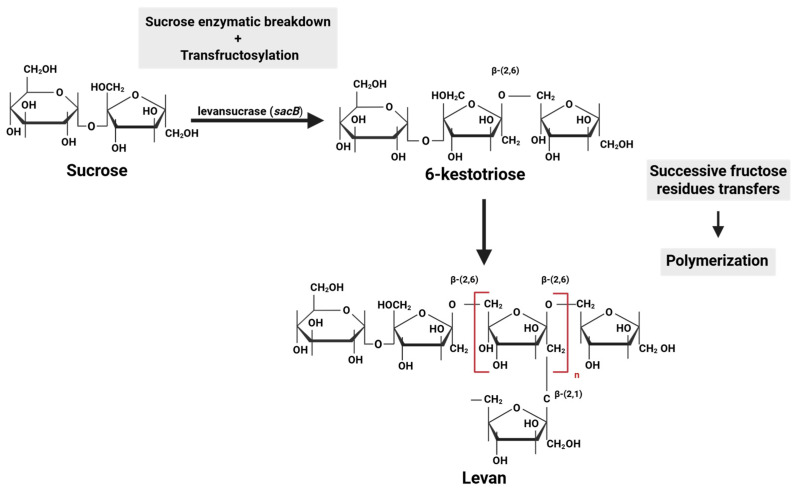
Microbial levan biosynthesis.

Microbial levan biosynthesis has three key steps: hydrolysis of sucrose from a sucrose-rich substrate, transfructosylation, and polymerization. All reactions are carried out by levansucrase, a fructosyltransferase belonging to the glycoside hydrolase family 68 (GH68). Thus, a sucrose molecule is cleaved by levansucrase into glucose and fructose. Then, a fructose molecule is transferred to a donor sucrose molecule, resulting in the formation of a levan-type trisaccharide, 6-kestotriose, by a bacterial levansucrase type sucrose–fructan 6-fructosyltransferase [73]. After successive fructose residue transfers, the polymeric chain elongates, forming levan molecules while releasing glucose molecules into the medium (polymerization) [66,67,74,75] (Figure 2).

The physico-chemical features of this biopolymer are conferred by its molecular architecture and depend on the source and production conditions [26]. It is soluble in both water and oil, but not in the vast majority of organic solvents, such as ethanol, methanol, isopropanol, toluene, or acetone [68]. Levan is a nonionic fructan with amphiphilic properties, possessing both hydrophilic and hydrophobic groups that allow polymer self-assembly into colloidal dispersions or nanoparticles. The assessment of stability in different pH conditions showed that acid hydrolysis occurs sooner at lower pH conditions, and no hydrolysis was observed for pH 5.5 and higher [76]. Levan has high thermal stability, with previous studies reporting a melting point at 214 °C for levan derived from *Bacillus subtilis* [58]. Thermal analysis of levan produced by *Acetobacter xylinum* showed several stages of thermal degradation. Increasing the temperature from 200 to 250 °C induced breakages in the β-(2,1) branch point linkages, followed by breakage of the main chain β-(2,6) linkages [68]. In spite of the high molecular weight, microbial levan has low intrinsic viscosity, ranging from 0.07 to 0.18 dL/g for polymers with molecular weights ranging from 16,000 to 24,000 kDa [77].

The aforementioned characteristics, such as solubility and viscosity, together with its adhesive, matrix-forming, water retention abilities, and compatibility with salts and surfactants, highlight levan as a promising candidate for pharmaceutical coatings and wound bandages [68,75]. Due to its amphiphilic properties, the production of levan-based nanoparticles has been explored to encapsulate hydrophobic materials. The amphiphilic nature of levan can be easily exploited for targeted drug delivery and tissue engineering [78,79]. The particular structure and physico-chemical features conferring thermal stability, biofilm-like matrix formation, and high biocompatibility define relevant functional properties for levan-based hydrogels [25].

### 3.1. Synthesis of Levan Hydrogels

The molecular and network structure of a hydrogel determines its fundamental nature and defines its functional properties and, therefore, performance in applications. Levan-based hydrogels can mimic the mechanical properties of the in vivo extracellular matrix and modulate the biological properties of host cells, thus being ideal candidates for biomedical purposes. Various synthesis protocols can be adapted for different types of hydrophilic gels with levan, ranging from natural to chemical and hybrid gels.

**Figure 3:**
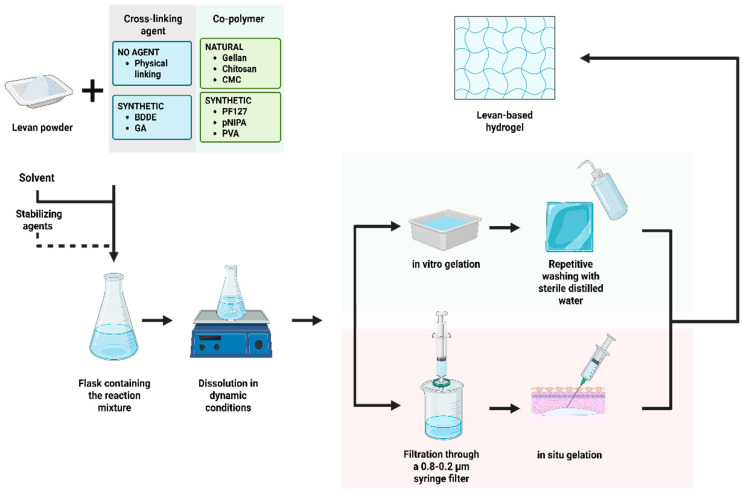
General steps for obtaining levan-based hydrogels. BDDE—1,4-butanediol diglycidyl ether; CMC—carboxymethyl cellulose; GA—glutaraldehyde; PF127—Pluronic F127; pNIPA—N-isopropyl acrylamide; PVA—polyvinyl alcohol.

The synthesis of levan-based hydrogels typically involves physical and/or chemical cross-linking. The process requires a series of successive steps for solubilization and purification of polymers, followed by a phase transition for gelation (Figure 3).

Initially, a levan powder is dissolved in dynamic conditions into a basic or neutral solvent, such as sodium nitrate [80], borate buffer [81], or deionized water [82,83]. If the aim is to produce a hybrid gel, an intermediary step is required for the solubilization of the co-polymer and the stabilizing agents into the same solvent [81,82,83].

The next step is the purification of the sol-state mixture. For standard hydrogels, purification consists of repetitive washing with sterile distilled water to remove impurities and/or contaminants [80]. For injectable hydrogels with delayed gelation, the process most commonly involves physical filtration through syringe filters ranging from 0.8 to 0.2 μm [79,82,83].

Lastly, the gel state is induced via different methods, depending on the components and the type of hydrogel. Phase transition for gelation is mostly induced via a thermal stimulus, which determines the interactions between hydrophilic and hydrophobic molecules. The introduction of a hydrophobic compound, such as a methyl or an ethyl group, requires a critical temperature, below the point when the mixture is miscible and above the value when phase separation occurs [6]. Thermo-chemical cross-linking is more suitable for obtaining levan-based hydrogels [84]. The process involves at least one cross-linking agent, such as 1,4-butanediol diglycidyl ether (BDDE) [71] or glutaraldehyde [85].

### 3.2. Design Strategies to Improve Levan-Based Hydrogels for Biomedical Purposes

**Figure 4:**
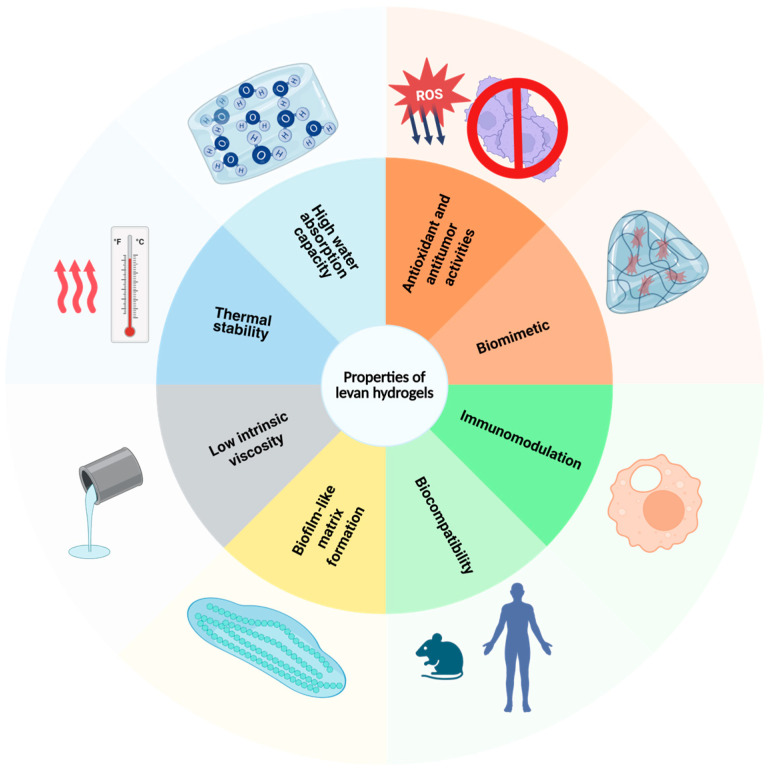
Properties of levan-based hydrogels for biomedical applications.

Hydrogel tailoring for specific uses allows us to control mechanical strength, swelling behavior, and degradation rates. Desired properties can be achieved by designing polymer composition, chemical modification and functionalization, gelation mechanism, and cross-linking agents. Therefore, a variety of options have been proposed to design levan-based hydrogels suitable for a plethora of biomedical applications, with antioxidant, antitumor, antibacterial, immunomodulatory, biofilm-like matrix formation, and biomimetic properties (Figure 4).

The antioxidant activity of levan-based hydrogels stems from the polymer’s strong scavenging abilities against free radicals and induction of anti-inflammatory cytokine production, while the antitumor capacity is a result of the modulation of host cell immune response via caspase activation and induction of apoptosis via the mitochondrial pathway [86,87,88]. Moreover, antibacterial properties are conferred by its polysaccharide backbone, capable of disrupting Gram-negative bacteria’s membrane integrity [89,90]. On top of that, levan-based hydrogels have been shown to possess biocompatible and biomimetic properties due to their porous networks, which favor cell adhesion and proliferation, alongside biomolecule and nutrient transport [91].

Hydrogel composition. Pure levan is not used alone in hydrogels due to inconsistent and difficult control of properties, whereas using levan derivatives or copolymers improves hydrogel properties and functional behavior. Levan methacrylate hydrogels were shown to produce cytocompatible, mechanically stable gels with a moderate degree of swelling. Semisynthetic levan derivatives contain methacrylate groups attached either via ester or urethane linkages to the fructan backbone. Levan methacrylates derived from *Bacillus subtilis* levan were photo-chemically cross-linked by applying different photoinitiator systems responsive to either UV light or visible light irradiation [81].

Chemical modification and functionalization. Chemically modified forms, such as hydrolized, sulphated, methylated, oxidized, and phosphonated levan derivatives, enable desirable properties of hydrogels, nanofibers, and films for medical applications [26]. Levan-based hydrogels often include copolymers, such as polyvinyl alcohol (PVA), poly-N-isopropyl acrylamide (pNIPA), or Pluronic F127 (PF-127). Levan–PVA hydrogels have a high water solubility and water adsorption ability [92]. Biocompatible methacrylated levan served as a cross-linker in the preparation of levan/pNIPA thermosensitive hydrogels [93]. Other experimental protocols have included PF-127 prepared with carboxymethyl cellulose (CMC) to improve its in vivo stability [82].

Gelation mechanism. Regarding gelation, there are several possibilities, with or without chemical interventions. For example, in the composite levan–gellan hydrogel, gelation occurs by increasing the pH to the alkaline domain, inducing interactions between gellan’s free hydroxyl groups by inter-linking with levan molecules [94]. Besides in vitro gelation through chemical agents, thermal inducers, or inducible polymerization, a specific biological cross-linking involves enzyme-mediated linkage, leading to in situ gelation of injectable hydrogels [79,81,83].

The influence of cross-linking agents. Most hydrogels are formed through cross-linking, either through physical or chemical methods. Physical methods imply linking via a stimulus and are usually reversible, while the rate of the inducer is used to modulate the final properties. Chemical cross-linking presents methods such as the formation of bonds through the addition of a cross-linking agent, polymer functionalization, radical polymerization, enzyme-mediated processes, and even irradiation [95]. Different agents and methods can therefore be used to influence and modify physico-chemical and rheological properties of polysaccharide-based gels, such as mechanical strength, stability, swelling, and water binding [85]. The available literature describes a variety of cross-linking protocols, but the specific influence of certain agents or techniques is insufficiently detailed. Therefore, this section aims to emphasize protocol designs with concrete examples of cross-linkers for particular applications (Table 2).

Chemical cross-linking. Chemical cross-linking may be achieved through a number of methods, most commonly through a chemical agent, photo-chemical reactions, or Schiff’s base reaction. The work by Selvi et al. [80] showed the influence of BDDE on both native and phosfonated *Halomonas*-derived levan hydrogels for resveratrol release, with the phosfonated derivative resulting in a firm, homogenous gel. However, an increase in BDDE raises the toxicity of the gels, despite the improvement in their stability and heterogeneity. Similar results were reported by Demirci et al. [96] regarding the stability of hydrogels synthesized for amphotericin B controlled release.

Another candidate is a biocomposite gel of oxidized levan–chitosan, with chitosan acting both as a cross-linking agent and a copolymer. Gel formation occurs through Schiff’s base reaction—oxidized levan aldehyde groups interact with the primary amines of chitosan, resulting in imine bonds, responsible for the three-dimensional gel architecture. The obtained hydrogel showed not only cytocompatibility but also hemocompatibility, besides good swelling capacities and stability [91].

Physical cross-linking. For physical cross-linkage, besides levan, a co-polymer is preferred for increased stability. Injectable hydrogels composed of levan, CMC, and PF127 have been synthesized as an alternative to other commercial dermal fillers, such as hyaluronic acid. CMC was selected to improve in vivo stability, and PF-127 was selected to enhance the cross-linking density by increasing the number of hydrogen bonds. Besides common in vitro testing, promising results on wrinkle mouse models have been obtained [82]. Another excellent compound triad is represented by levan–PVA gels cross-linked with glutaraldehyde (GA) for influenza virus capture. GA and PVA increase the stability and strength of pure levan gels by forming acetal bridges when incubated at room temperature, followed by freeze-drying [92].

### 3.3. Strategies to Improve Rheological Properties of Levan-Based Hydrogels for Biomedical Purposes

Levan is an unusual carbohydrate, a water-soluble polymer with native properties ranging from biocompatibility and health benefits due to its distinctive rheological features [94]. Rheological testing is often an indispensable tool for analyzing specific characteristics of hydrophilic gels, such as the deformation and flow [99]. Some relevant aspects for biomedical engineering, but not limited to levan-based hydrogels, are swelling, capacity, viscoelasticity, tensile strength, and macroscopic behavior.

Swelling capacity. The consensus regarding the influence of a cross-linker on the swelling ability of a gel is that a high concentration of the cross-linking agent leads to higher-density gels and, thus, a lower water absorption capacity; this behavior applies to levan hydrogels as well [96]. Besides that, for most hydrophilic gels, it is common knowledge that adding an ionic monomer or increasing the number of ionic groups, for example, the addition of salts, increases the swelling degree. This is caused by the presence of a high number of counter-ions, leading to additional osmotic pressure, a pH-independent process, and thus inducing a stimulus-independent behavior [7], and it can be speculated to be applicable to levan hydrogels as well.

Viscoelasticity. Gels are viscoelastic systems and, therefore, possess the capacity for shear thinning and/or thickening. However, they are shear-thinning by default due to a high fluid content. There have been reports of hydrogels based on recombinant proteins or peptides, thus non-polysaccharides, which are capable of shear-thinning and self-healing, though they present disadvantages such as poor mechanical properties and cellular and in vivo toxicity. Hence, biocompatible alternatives with improved stability have been sought through natural carbohydrate-based polymers, including composite gels composed of levan and gellan [94].

Tensile strength. Molecular branches may contribute to the cohesive/adhesive properties of levan [99], while the presence of hydroxyl groups facilitates its interaction with various molecules [95]. Moreover, using levan derivatives has shown good improvements regarding tensile strength, a property in direct relation to shearing. For example, a desirable increased tensile strength of sulfated levan hydrogels, combined with alginate and chitosan, led to the development of both chemically cross-linked and non-linked gels in adhesive-free standing multilayer films for biomedical purposes [99,100].

## 4. Current and Potential Biomedical Applications of Levan-Based Hydrogels Based on Their Properties

This section aims to describe a few of the current applications associated with levan-based hydrogels. The literature has vast examples, studies, and papers on exopolysaccharide gels; however, only a few focus on levan hydrogels specifically, and their potential is still not fully explored. Compiling all the available information confirms that levan hydrogels and levan-based colloidal systems are promising candidates for the biomedical field [101], with applications ranging from targeted drug delivery/therapeutic agents to regenerative medicine, wound healing, tissue engineering, and even bioprinting ink.

Targeted drug delivery systems. Studies on levan hydrogels as delivery systems for therapeutic substances, such as amphotericin B [96], vancomycin [102], or resveratrol [80], indicate promising results, without affecting the bioavailability of the therapeutic agent. However, there is much more to explore concerning both the hydrogel composition and the form in which the gel is used. Hydrogel-based drug delivery systems are generally represented by nanoparticles [103].

The exciting possibilities of levan nanocarriers also include protein and chemotherapeutic agent delivery systems. Sezer et al. [104] investigated the in vitro release of bovine albumin serum, cementing their suitability as drug carriers. Besides that, levan has an affinity for binding to CD44 receptors. Thus, the potential of paclitaxel-loaded levan nanoparticles has been explored to efficiently deliver chemotherapeutic agents to cancer cells [105]. Moreover, the efficacy of levan–poly (lactic-co-glycolic acid) nanoparticles has been exploited for their delivery of chemotherapeutic and chemopreventive agents [106].

Wound healing and rejuvenation. Other interesting aspects related to the possible applications of levan-based hydrogels are wound healing and the process of rejuvenation. Hydrogels, mainly carbohydrate-based gels, have already been characterized as wound dressing materials due to their non-antigenicity and permeability to water and metabolites whilst isolating the wound site from pathogens [107]. Previous investigations focused on microbial levan as an adjuvant to accelerate the healing of burn injuries. The effects are possibly achieved through the activation of matrix metalloproteinase enzymes, which are crucial during the repair of damaged tissues [108]. Hence, their incorporation in a hydrogel system may be a potential approach for wound treatment. Rejuvenation of soft tissue was also achieved through an injectable levan-based hydrogel, with improved biocompatibility and good in vitro and in vivo stability in comparison to hyaluronic acid hydrogels [82]. Similarly, Hwang et al. improved on the previous hydrogel formulation by adding hydroxyapatite, thus increasing its stability and residence time while keeping the original features, such as biocompatibility and collagen production stimulation [83].

Tissue engineering and bioprinting. Polysaccharide hydrogels have also been investigated for their applications in tissue engineering due to their tridimensional structure and biomimetic abilities, especially in conjunction with cellular matrices, via in situ scaffold formation [109]. By combining both bioprinting technology and tissue engineering, the use of methacrylate levan, a photocurable variety, as bio-ink for bone tissue scaffolds and for a final construct containing pre-osteoblasts, led to favorable cell proliferation and subsequent osteogenesis. This levan-based hydrogel bio-ink formulation highlights its immunomodulatory properties via modulating macrophage phenotype and promoting expression of anti-inflammatory markers [98]. For guided bone regeneration, histopathological studies indicated that the combination of levan hydrogels and conventional bone graft materials led to improved osteoblast formation and neovascularization, rather than the use of deproteinized bovine grafts alone [97]. On top of that, sulfated levan thin-layer films have been investigated for cardiac tissue engineering, not only for their heparin-mimetic capacity, but also for their high rate of functionalization [110]. In the same vein as levan thin-layer films, the current literature has investigated adhesive free-standing multilayer films containing sulfated levan, which promote both myogenic differentiation and mioconductivity [100]. In addition, ternary blend films of chitosan/polyethylene oxide/levan promoted cell proliferation and viability [111], thus pertaining to tissue engineering and bioprinting applications.

## 5. Perspectives and Challenges

Levan-based hydrogels are still an underexplored frontier because of current experimental limitations. For example, levan-based hydrophilic gel matrices could incorporate not only pre-osteoblasts [97] but also other cell types, which are already incorporated into polysaccharide-based hydrogels: the COS-7 cell line, NIT 3T3 fibroblasts [112], cortical neurons isolated from chick embryos [113], and chondrocytes isolated from porcine cartilages [114]. This leads to the field of bioprinting, where hydrogels are the bio-ink used for three-dimensional cell matrices. However, there are still obstacles related to their optimization in order to accommodate different cell types, specifically maintaining structural fidelity over time [115], as well as a lack of dynamism, which directly impacts biomimicry [116]. Moreover, improvement in mechanical strength by increasing either the polymer’s or cross-linking agent’s concentration may compromise the bioactivity of certain molecules, consequently limiting their biological applications [117]. On top of that, the aspect of uneven and/or cross-linking impacts their mechanical, physico-chemical, and biological activities, requiring additional post-processing [118], such as the inclusion of rheology modifiers [119].

An additional area with tremendous potential would be the development of biosensors and resorbable electronics. The previous literature does not address levan in the hydrogel system, but in different constructs, like a modified levan sensor proved suitable for the voltametric detection of daunorubicin–DNA interactions [120]. Kwon et al. described the use of levan-based films for transient electronic systems, with promising results at both in vivo and in vitro testing, concerning biocompatibility and bioresorption [121].

Unfortunately, creating functional levan hydrogels poses another set of challenges in addition to those mentioned above. Another limitation is the spatial inhomogeneity in hydrophilic gels, caused by high concentrations of the monomer unit and the concentration of polymers at the level of the linking region. However, it can be managed through temperature and concentration adjustments, or incorporation of synthetic components, to achieve stabilized and uniform cross-linked networks [7,10]. Nevertheless, the addition or increase in concentration of a synthetic component may lead to toxicity issues [80,82]. A fundamental problem of polymeric materials resides in the precise control of the polymer structure [122], with particular importance in the context of bioinspired materials. Finally, an obstacle that is not related to the technical side itself is represented by the scalability and reproducibility of these gels for commercialization purposes, implicitly, the approval by regulatory agencies [123].

## Figures and Tables

**Table 1:** Bacteria as sources of levan and levansucrase.

Lineage: Phylum, Class, Order, Family	Species	Strain	Source	References
Actinomycetota; Actinomycetes; Micrococcales; *Dermabacteraceae*	*Brachybacterium phenoliresistens*	n.a.	Rhizosphere	[36]
Actinomycetota; Actinomycetes; Micrococcales; *Microbacteriaceae*	*Microbacterium laevaniformans*	PTCC 1406	Active sludge	[37]
Actinomycetota; Actinomycetes; Micrococcales; *Microbacteriaceae*	*Microbacterium* sp.	XL1	Soil	[38]
Bacillota; Bacilli; Bacillales; *Bacillaceae*	*Bacillus amyloliquefaciens*	n.a.	n.a.	[39]
Bacillota; Bacilli; Bacillales; *Bacillaceae*	*Bacillus lentus*	V8	Rhizosphere	[40]
Bacillota; Bacilli; Bacillales; *Bacillaceae*	*Bacillus licheniformis*	BK AG21	Mud crater	[41]
Bacillota; Bacilli; Bacillales; *Bacillaceae*	*Bacillus megaterium*	PFY-147	Rhizosphere	[42]
Bacillota; Bacilli; Bacillales; *Bacillaceae*	*Bacillus methylotrophicus*	SK 21.002	Soil	[43]
Bacillota; Bacilli; Bacillales; *Bacillaceae*	*Bacillus mojavensis*	n.a.	Soil	[44]
Bacillota; Bacilli; Bacillales; *Bacillaceae*	*Bacillus paralicheniformis*	n.a.	Rhizosphere	[45]
Bacillota; Bacilli; Bacillales; *Bacillaceae*	*Bacillus subtilis*	HMNig-2; MENO2 AF17	Honey Honey bee gut Kefir	[46] [47] [48]
Bacillota; Bacilli; Bacillales; *Paenibacillaceae*	*Paenibacillus polymyxa* (formerly *Bacillus polymyxa*)	n.a.	Soil	[49]
Bacillota; Bacilli; Bacillales; *Paenibacillaceae*	*Paenibacillus* sp.	#210	Crude oil	[50]
Bacillota; Bacilli; Lactobacillales; *Lactobacillaceae*	*Leuconostoc citreum*	BD1707	Kefir	[51]
Bacillota; Bacilli; Lactobacillales; *Lactobacillaceae*	*Limosilactobacillus reuteri* (formerly *Lactobacillus reuteri*)	FW2	Fish gut	[52]
Pseudomonadota; Alphaproteobacteria; Acetobacterales; *Acetobacteraceae*	*Gluconobacter albidus*	TMW 2.1191	Water kefir	[53,54]
Pseudomonadota; Alphaproteobacteria; Acetobacterales; *Acetobacteraceae*	*Gluconobacter japonicus*	LMG 1417	n.a.	[55]
Pseudomonadota; Alphaproteobacteria; Acetobacterales; *Acetobacteraceae*	*Komagataeibacter xylinus* (formerly *Acetobacter xylinum*)	NCIM 2526	n.a.	[56]
Pseudomonadota; Alphaproteobacteria; Sphingomonadales; *Zymomonadaceae*	*Zymomonas mobilis*	NRRL B-14023	Sugarcane fermentations	[57]
Pseudomonadota; Gammaproteobacteria; Enterobacterales; *Erwiniaceae*	*Erwinia amylovora*	n.a.	n.a.	[58]
Pseudomonadota; Gammaproteobacteria; Enterobacterales; *Erwiniaceae*	*Pantoea agglomerans*	ZMR7	Rhizosphere	[59]
Pseudomonadota; Gammaproteobacteria; Enterobacterales; *Pectobacteriaceae*	*Brenneria goodwinii*	OBR1	n.a	[60]
Pseudomonadota; Gammaproteobacteria; Moraxellales; *Moraxellaceae*	*Acinetobacter nectaris*	CECT 8127	Floral nectar	[61]
Pseudomonadota; Gammaproteobacteria; Oceanospirillales; *Halomonadaceae*	*Chromohalobacter japonicus*	BK-AB18	Mud crater	[62]
Pseudomonadota; Gammaproteobacteria; Oceanospirillales; Halomonadaceae	*Halomonas elongata*	BK-AB8; BK-AG18; 153B	Mud crater Saltern	[62] [63]
Pseudomonadota; Gammaproteobacteria; Oceanospirillales; *Halomonadaceae*	*Halomonas eurihalina*	BK-AB15	Mud crater	[62]
Pseudomonadota; Gammaproteobacteria; Oceanospirillales; *Halomonadaceae*	*Vreelandella meridiana* (formerly *Halomonas meridiana*)	BK-AB4	Mud crater	[62]
Pseudomonadota; Gammaproteobacteria; Oceanospirillales; *Halomonadaceae*	*Halomonas smyrnensis*	AAD6^T^	Saltern	[64]
Pseudomonadota; Gammaproteobacteria; Pseudomonadales; *Pseudomonadaceae*	*Pseudomonas fluorescens*	NCIM 2059	n.a.	[65]

Table legend: n.a. = not available; NCIM = National Collection of Industrial Microorganisms; NRRL = Northern Regional Research Laboratory; PTCC = Persian Type Culture Collection.

**Table 2:** Examples of cross-linking types and their applications.

Cross-Linking Agent/Co-Polymer	Cross-Linkage Type	Levan Source	Applications	Ref.
PF127/CMC	Physical (thermo-cross-linking)	X—not mentioned, commercially available levan powder	Injectable dermal filler alternative: stimulation of dermal fibroblast proliferation and collagen production. Soft tissue regeneration. Anti-wrinkle effects.	[82]
GA/PVA	Chemical	X—not mentioned, commercially available levan powder	Influenza A virus adsorption from aqueous solutions. Levan–PVA filters capable of successfully capturing bioaerosol samples containing the virus.	[91]
PF127/CMC/Hydroxyapatite composite	Physical (thermo-cross-linking)	*Erwinia herbicola*	Long-acting/semi-permanent dermal filler/anti-wrinkle effects. Improved dermal fibroblast proliferation and collagen production. Enhanced stability/in vivo residence.	[83]
BDDE	Chemical	*Halomonas smyrnensis* AAD6^T^	Therapeutic agent delivery: amphotericin B for dermal antifungal treatment against *Candida albicans*.	[96]
Levan/pNIPA	Physical (thermo-cross-linking)	*Halomonas smyrnensis* (from bioreactor cultures)	pH-responsive drug delivery system with improved biocompatibility for 5-aminosalycylic acid.	[93]
BDDE	Chemical	*Halomonas smyrnensis* (from bioreactor cultures)	Native and phosphonated levan hydrogels for entrapment and controlled release of resveratrol. Improvement in bovine bone graft materials, through increased osteoblast formation and vascularization, with promising in vitro and in vivo results.	[80,97]
UV photo-initiators (I 2959/LAP)	Physical (photo-cross-linking)	*Bacillus subtilis*	Levan methacrylate-based gels for possible biomedical applications due to cytocompatibility determined via live/dead assay.	[81]
Chitosan/oxidized levan composite	Chemical (Schiff’s base reaction)	*Bacillus subtilis* MTCC 441	Improving drug release of chemotherapeutic and chemopreventive agents (curcumin). Promising wound dressing material due to hemocompatibility and cytocompatibility.	[91]
Levan–gellan composite	Chemical	*Erwinia herbicola* L8647	Biocompatible and biodegradable novel injectable material in the medical/cosmetic industry.	[94]
UV photo-initiator (LAP)	Physical (photo-cross-linking)	*Bacillus* sp. SGD-03	Levan–methacrylate hydrogel as bio-ink for bone tissue engineering. Modulation of macrophage phenotype and promotion of anti-inflammatory markers.	[98]

LAP—lithium phenyl (2,4,6-trimethyl benzoyl) phosphonate.

## Data Availability

No new data were created or analyzed in this study. Data sharing is not applicable to this article.
